# A screening approach for assessing lytic polysaccharide monooxygenase activity in fungal strains

**DOI:** 10.1186/s13068-019-1526-4

**Published:** 2019-07-22

**Authors:** Pooja Dixit, Biswajit Basu, Munish Puri, Deepak Kumar Tuli, Anshu Shankar Mathur, Colin James Barrow

**Affiliations:** 1 DBT-IOC Centre for Bioenergy Research, R&D Centre, Indian Oil Corporation Limited, Faridabad, India; 2 School of Life and Environmental Sciences, Deakin University, Geelong, VIC Australia; 3 Centre for Marine Bioproducts Development, College of Medicine and Public Health, Flinders University, Adelaide, Australia

**Keywords:** AA9 LPMOs, *Penicillium* sp. LPMOs, Synergy in cellulases, Cellulose, Hemicellulose, Acid-treated rice straw, UPLC–ESI–MS, Gluconic acid, Cellobionic acid, Biorefinery

## Abstract

**Background:**

Efforts to develop efficient lignocellulose-degrading enzymatic preparations have led to the relatively recent discovery of a new class of novel cellulase boosters, termed lytic polysaccharide monoxygenases (LPMOs). These enzymes are copper-dependent metalloenzymes that initiate the biomass deconstruction process and subsequently work together with cellulases, hemicellulases, and other accessory enzymes to enhance their hydrolytic action. Given their wide distribution and diversity, screening and isolation of potent LPMOs from natural fungal diversity may provide an important avenue for increasing the efficiency of cellulases and thereby decreasing cellulosic ethanol production costs. However, methods for quick screening and detection are still not widely available. In this article, a simple and sensitive method is described by combining nonhydrolytic activity enhancement followed by LC–MS-based quantitation of LPMOs.

**Results:**

In this study, a screening approach has been developed for the detection of nonhydrolytic cellulase-enhancing enzymes in crude fungal supernatants. With the application of a saturating benchmark cocktail of Celluclast 1.5L, fungal isolates were selected which had the capability of hydrolyzing pretreated rice straw by their synergistic enzyme fractions. Subsequently, these fungal extracts along with an LPMO-enriched commercial enzyme were investigated for their ability to produce Type I LPMO activity. An LC–MS-based methodology was applied to quantitate gluconic acid in enzymatic hydrolysates as an indirect measurement of Type I LPMO activity.

**Conclusion:**

The present study describes an LC–MS-based separation method to detect and quantitate LPMO activity in a commercial enzyme. This method was also applied to screen fungal extracts. The developed screening strategy has enabled detection of LPMO activity in two industrially important *Penicillium* strains.

**Electronic supplementary material:**

The online version of this article (10.1186/s13068-019-1526-4) contains supplementary material, which is available to authorized users.

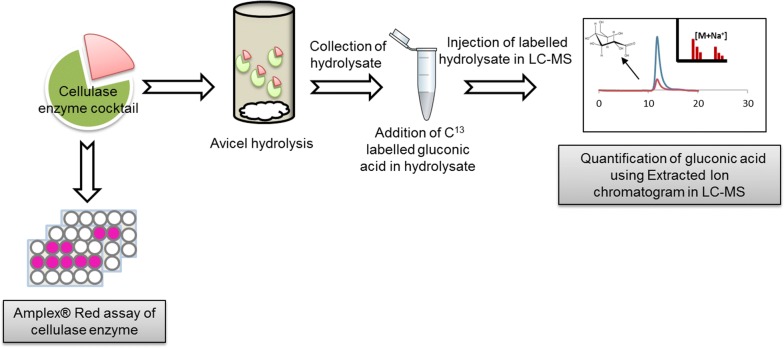

## Background

The goal of advancing toward a biobased fuel economy has popularized the concept of biorefinery, which includes the efficient and sustainable utilization of lignocellulosic biomass for energy building purposes [1]. The energy reserves of lignocellulosic biomass are mainly stored in C5 and C6 carbon sugar polymers (cellulose and hemicellulose), while the aromatic heteropolymer lignin is a renewable precursor for carbon materials and energy-storage devices and is being investigated for its commercial-level valorization. Enzymatic saccharification unlocks the energy potential of lignocellulosic sugar polymers by employing an arsenal of cellulases, hemicellulases, and other accessory enzymes [2].

Despite extensive research focused on enzyme development processes, there is no superior single enzymatic system that can provide cost-effective and satisfactory enzymatic hydrolysis on different lignocellulosic biomasses [3]. The need to develop more efficient and effective enzymatic preparations has led to the application of sophisticated system biology tools that allow researchers to identify and develop novel and efficient enzymatic components. The application of these powerful tools has led to the discovery of novel nonhydrolytic proteins such as lytic polysaccharide monooxygenases (LPMOs), swollenins, and loosenins which are reported as “synergistic enzymes” that boost cellulase action [4].

Lytic polysaccharide mono-oxygenases (LPMOs) are copper-dependent metalloenzymes that initiate the biomass deconstruction process and subsequently synergize with enzymes such as cellulases to enhance their hydrolytic action on a range of polysaccharides, including cellulose [5]. Currently LPMO spans five different classes in the CAZY database as “Auxiliary Activity” proteins. Fungal LPMOs are AA9 enzymes that primarily act on cellulose. Chitin- and cellulose-oxidizing bacterial candidates are AA10 enzymes, while the AA11 class of LPMOs consists of fungal LPMOs that act on chitin, AA13 enzymes oxidizes starch, and lastly AA14 class of LPMOs which target xylan degradation [6–9]. All LPMOs contain a conserved histidine brace containing a copper center at their active site, which mediates an electron transport chain to cleave glycosidic bonds. LPMOs oxidize the sugar carbon in cellulose chains at either the C1 or C4 position, resulting in the formation of oxidized as well as reduced glucose monomers as hydrolysis end products [10]. Depending upon their point of action, LPMOs are classified as: Type 1 that oxidizes only at the C1 carbon in cellulose chain to yield aldonic acids, or Type 2 that act upon the C4 carbon to form nonreducing end C4-ketoladose, which are generally found in equilibrium with gemdiols in aqueous conditions. Lastly Type 3 act on both C1 and C4 carbon atoms in the cellulose chain. This oxidative cleavage requires the presence of redox cofactors that can donate electrons to LPMO copper centers. A plethora of such LPMO essential redox molecules have been reported, which include small reductants such as ascorbate, gallate, lignin-derived aromatic biomolecules, photosynthetic pigments, and co-secreting AA3 family GMC oxidoreductases (cellobiose dehydrogenase, glucose dehydrogenase etc.) [11].

The concerted action of LPMO on its natural insoluble substrate and the dynamic interaction of LPMO with its redox-active cofactors are important mechanistic questions that are still unresolved. To determine mechanisms of action, analytic methods such as high-performance anion-exchange chromatography-pulsed amperometric detector (HPAEC-PAD), Liquid chromatography-mass spectrometry (LC–MS) and matrix-assisted laser desorption/ionization–time of flight-mass spectrometry (MALDI-TOF–MS) have been applied to characterize oxidized LPMO products. With the goal of finding an efficient and sensitive method for the separation of native and oxidized cello-oligosaccharides, Westereng et al. [12] evaluated different HPLC-based analytic tools and concluded that HPAEC-PAD provided a superior and sensitive LC-based quantitation method for the oxidized species. The authors suggested that LC–MS-based approaches such as hydrophilic interaction chromatography (HILIC-MS) or porous graphitized carbon liquid chromatography (PGC-LC–MS) are particularly useful, with PGC-LC–MS being the method of choice for simultaneous analysis of C1 and C4 oxidation products.

There is no robust assay protocol for evaluating LPMO activity of natural microbial diversity. There are some reports where a colorimetric Amplex^®^ Red assay has been used for quantitating LPMO activity in broth [13]. However, due to its nonspecific nature, this assay can only be applied for quantitating the total H_2_O_2_ production potential of strains and not LPMO activity. Detection and quantitation of oxidized cellulose hydrolysis products (i.e. gluconic acid and cellobionic acid) can be used as an indirect way to measure LPMO activity in cellulases. There are only a few reports that have characterized the oxidative activity of Cellic™ Ctec2 by measuring both C1- and C4-oxidized cellodextrins produced after cellulose hydrolysis, using HPAEC-PAD [14–16].

HPAEC-PAD has been the most widely utilized tool for analyzing cello-oligosaccharides and their oxidation products; however, this method has limitations including sensitivity. It has been reported that HPAEC-PAD method can be used to quantitate gluconic acid solution up to 50 ppm in concentration [14]. For the quantitation of oxidized species produced at the sub-ppm levels, as anticipated from natural microbes, a more sensitive method such as LC–MS might be beneficial. Therefore, the present study utilizes an LC–MS-based method to monitor gluconic acid concentrations in enzymatic hydrolysates as an indirect measure of LPMO activity in fungal enzymes. Another objective of this study was to develop a robust screening protocol of fungal enzymes for their AA9-type LPMO production. The developed protocol was then applied to the screening of two industrially important *Penicillium* species.

## Materials and methods

### Chemicals and commercial enzymes

All the chemicals used in this study were procured from Sigma Aldrich and Merck, India.

^13^C gluconic acid (99 atom % ^13^C) were purchased from Omicron Biochemicals, USA, and LC/MS grade water was obtained from Biosolve Chimie, SARL, France. Commercial enzymes Cellulase from *T. reesei* ATCC 26921 (Celluclast 1.5L) and β-glucosidase (Novozymes 188) were procured from Sigma, Aldrich India. Cellic^®^ Ctec2 and Cellic^®^ HTec3 were a kind gift from Novozymes, Mumbai, India. Avicel^®^ PH 101 and dilute acid-treated rice (*Oryza sativa)* straw were used as substrates for enzymatic saccharification studies. Rice (*Oryza sativa)* straw was procured from the local market at Mathura (27.28° N, 77.41° E) in Uttar Pradesh (North, India) air-dried, ground to the particle size of ~ 10 mm using a knife mill ,and stored in an air tight container for further use. All the experiments were conducted from the single stock of harvested rice straw.

### Strains

*Penicillium janthinellum* (NCIM 1171) and *Penicillium funiculosum* (NCIM 1228) were procured from the National Collection of Industrial Microorganisms (NCIM) Pune, India. All fungal strains examined in this study were derived by UV-irradiation and chemical mutagenesis of the above-mentioned fungal species. The resulting mutants were selected based on relative enzyme index on amorphous cellulose (I_AC_) and were maintained and stored in Potato Dextrose agar (PDA) slants at 4 °C until further use.

$${\text{Relative}}\,{\text{enzyme}}\,{\text{index = }}\frac{{{\text{Diameter}}\,{\text{of}}\,{\text{zone}}\,{\text{of}}\,{\text{clearance}}\,{\text{on}}\,{\text{amorphous}}\,{\text{cellulose plate by mutant}}}}{{{\text{Diameter}}\,{\text{of}}\,{\text{zone}}\,{\text{of}}\,{\text{clearance}}\,{\text{on}}\,{\text{amorphous}}\,{\text{cellulose plate by parent}}}}$$

### Production of in-house enzymes

The inoculum was prepared for all fungal cultures by inoculating 1 × 10^7^ fungal spores in seed media consisting of (NH_4_)_2_·SO_4_ (1 g/L), MgSO_4_ (0.2 g/L), CaCO_3_ (0.5 g/L), sucrose (10 g/L), corn steep liquor (5.4 g/L), Avicel (2 g/L), and Tween 80 (1 g/L). After 48 h, when the packed cell volume of culture reached around 30–40%, second inoculation was done in production media which consisted of (NH_4_)_2_·SO_4_ (5 g/L), MgSO_4_ (0.2 g/L), CaCO_3_ (0.5 g/L), corn steep liquor (5.4 g/L), Avicel (33 g/L), and Tween 80 (1 g/L). The culture broths/secretome were harvested at the 96th h of fermentation by centrifugation at 6000 rpm for 15 min to obtain crude cellulase enzyme, which was stored at 4 °C until further use.

### Enzymatic assays

All the hydrolytic enzymatic assays of crude fungal supernatants were performed in sodium citrate buffer (50 mM, pH 5.0) at 50 °C. The substrate hydrolysis assays for filter paper (FPU), carboxy methyl cellulose (CMC), and birch wood xylan were done by DNSA method, as described earlier [17].

Filter paper activity was determined by adding crude fungal extract (100 μL) in citrate buffer (1900 μL) containing Whatman no. 1 filter paper (1 × 6 cm strip; 50 mg), after which the reaction mixture was incubated at 50 °C for 60 min. Endoglucanase (CMCase) and *endo*-1,4-β-d-xylanase activity assays were carried out by adding 100 µL of appropriate enzyme dilution in 900 µL of 1% CMC and 1% birch wood xylan solution, respectively. The reaction mixtures were incubated at 50 °C for 30 min to carry out the respective substrate hydrolysis reactions. All the above-mentioned reactions were terminated by adding 3 mL of 3–5, Dinitrosalicylic acid (DNS) reagent. The reaction mixtures were boiled for 5 min, and the enzyme quantitations were done by measuring OD at 540 nm using standard glucose stock (10 mg/mL).

β-Glucosidase activity was estimated using *p*-nitro phenyl-α-glucopyranoside (*p*NPG) as substrate. 900 µL of substrate solution pNPG (1 mg/mL) was incubated with 100 µL of fungal enzyme at 50 °C for 30 min. The reactions were stopped by adding 2 mL of sodium carbonate (2%), and the liberated *p*-nitrophenol was quantitated at 410 nm using a *p*-nitrophenyl standard curve. One unit (IU) of enzymatic activity was defined as the amount of enzyme required to liberate 1 µmol of glucose, xylose, or *p*-nitrophenol from the appropriate substrates/min of crude fungal supernatant under the assay conditions.

Lytic polysaccharide monooxygenases (LPMOs) activity of crude fungal extracts were analyzed by following the previously reported method [13]. 20 μL of fungal supernatant was incubated with 180 μL of assay cocktail containing 300 μM ascorbate, 500 μM Amplex^®^ Red, and 71.4 units/mL horseradish peroxidase (HRP). The reactions were carried out in 100 mM sodium phosphate buffer, pH 6.0 at 22 °C, and the absorbance was measured at 560 nm after 10-min incubation using a plate reader (Spectra Max M3, Molecular Devices, USA). The reactions were also carried out in the presence and the absence of different sugars (glucose and cellobiose, final concentrations 500 μM). Specific LPMO activity is defined as one µmol H_2_O_2_ generated per minute per mg protein of crude fungal extracts, under the defined assay conditions.

Cellobiose dehydrogenase (CDH) activity of fungal enzymes was determined by lactose-mediated reduction of 2,6-dichloroindophenol (DCIP) at 30 °C, and absorbance was measured at 520 nm wavelength (extinction coefficient ε520 = 6.80 mM^−1^ cm^−1^) [18]. 1 mL reaction mixture contained 300 µM DCIP and 30 mM lactose in 100 mM sodium acetate buffer at pH 4. To suppress the laccase activity, sodium fluoride was added in reaction mixtures which would otherwise interfere with CDH measurements. One unit of CDH activity catalyzes the oxidation of 1 μmol of lactose per minute under the specified assay conditions.

Protein content of enzymes was measured using commercial BCA kit (Alfa Aesar, India) with BSA as standard.

### Dilute acid pretreatment of rice straw at pilot scale

Pretreatment of rice straw was done in pilot scale plant (250 kg/day capacity) through a two-step procedure as reported previously [19]. Initially rice straw was soaked in 0.4% alkali solution for 1 h followed by dilute acid (1% H_2_SO_4_) soaking of rice straw in a soaking chamber. After soaking, the wet biomass was drained and pressed with the help of a hydraulic filter press for 15 min at 100 bar. After this preprocessing, biomass was loaded to a reactor and pretreated at a temperature of 162 °C at 5.4 bar pressure with a residence time of 10 min. Following pretreatment, the biomass slurry was collected in slurry tank, allowed to cool, and then neutralized with a 30% ammonium hydroxide solution. With the help of peristaltic pumps, neutralized slurry was loaded to a high speed centrifuge to separate solids (cellulose and lignin) and liquid (hydrolyzed hemicellulose) fractions of the pretreated material. The solid residue of pretreated rice straw was washed several times with distilled water, followed by a last wash with sodium citrate buffer (50 mM, pH 5.0), and then stored at − 20 °C for further use. All the experiments were conducted with this single lot of washed pretreated rice straw.

The chemical composition of pretreated solid residue as well as of the native rice straw was determined by two-stage acid hydrolysis as per the standard NREL/TP-510-42618 protocol [20]. Sugar analysis was conducted with the help of Waters HPLC (Switzerland) equipped with Aminex HPX-87H column (Bio-Rad Laboratories, CA, USA) connected to a guard column. Sulfuric acid (0.008 N) was used as mobile phase at a flow rate of 0.6 mL/min, with a column temperature of 50 °C. Sugars (glucose, xylose, cellobiose, and arabinose) were analyzed with the help of Refractive Index detector and other inhibitory compounds (HMF and furfural) by a UV detector.

### Enzymatic hydrolysis

Hydrolysis was carried out at 10% (w/v) solids loading in 50 mM sodium citrate buffer (50 mM, pH 5.0). A total of 20 mL of reaction mixture containing 2 g of substrates (dry weight basis) and 0.02% sodium azide was dispersed in 250 mL of Erlenmeyer flasks. The enzymatic saccharification reactions were carried out at 50 °C for 48 h, under shaking conditions (200 rpm).

The enzyme dosing in the hydrolysis reactions was done according to the different experimental setup conditions. Firstly, pretreated rice straw was hydrolyzed with 7 FPU/g biomass Celluclast 1.5L in the presence and the absence of various concentrations of β-glucosidase (Novozymes 188) and a xylanase enzyme (Cellic Htec ^®^). The saturated concentration was 7 FPU of Celluclast 1.5L, 21 U of β-glucosidase, and 500 U of xylanase, which was used as control reaction. After saturation of Celluclast 1.5L, fungal secretomes were spiked in saturated cocktail of Celluclast 1.5L, and hydrolysis of pretreated rice starw was carried out with the formulated fungal-Celluclast blends. In this experimental set, 3.5 FPU of Celluclast was mixed with 3.5 FPU of fungal extracts, plus 21 U of β-glucosidase, and 500 U of xylanases, and the hydrolysis of pretreated rice straw was carried out.

After screening, the pretreated rice straw was hydrolyzed with 7 FPU of crude enzyme extracts from selected *Penicillium* sp. fungal candidates. This reaction was carried out to measure gluconic acid in rice straw hydrolysate and Cellic Ctec 2 (7 FPU/g) was used as the control in this reaction. Later, Avicel hydrolysis was carried out with a cellulase dosing of 7 FPU/g substrate with saturating β-glucosidase concentrations (21 U of Novo 188/gm substrate), in the presence and the absence of 1 mM ascorbate.

Samples were withdrawn at various intervals, boiled for 10 min to stop the reaction, and filtered through 0.45 µm filter to quantitate sugars as described in the above section.

### Product analysis of gluconic and cellobionic acids by LC–MS

LC was performed on a Thermo Scientific Ultimate 3000 UHPLC system (Thermo Fisher Scientific, MA, USA) equipped with Bio-Rad HPX-87H column (Bio-Rad Laboratories, CA, USA) operated at 50 °C. The solutes were eluted using a mobile phase of water containing 0.5% formic acid at a flow rate of 0.6 mL/min. Formic acid was preferred over conventional sulfuric acid due to lesser corrosion in spray chamber and spray shield of the electro spray mass spectrometer without any loss of resolving power.

The LC was interfaced with Q–Q–TOF ESI–MS (Bruker Impact II) from Bruker Daltonik, Germany. The mobile phase from column outlet was split in 1:4 ratio and the lowest flow (0.12 mL/min) was directed to the mass spectrometer. The analytes were monitored as sodium adduct, and a syringe pump was used for post column doping of eluent with 30 mM of sodium chloride solution in water at a flow rate of 60 µL/h through a T-split immediately before ESI source. ESI–MS was operated in positive ion mode and tuned for gluconic acid (capillary voltage, 4.5 kV; nebulizer, 1.4 bar; dry gas, 11.0 L/min, dry temperature, 250 °C, mass range, *m/z* 50–700).

The samples analyzed during the work were enzymatic hydrolysates containing cellobiose, glucose, and xylose, of which glucose was the major constituent. It was observed that glucose beyond 1000 ppm significantly suppressed gluconic acid response due to matrix effects (data not shown). Therefore, the enzymatic hydrolysates were diluted 50 times to ensure the concentration of glucose was below 1000 ppm and the injection volume was 5 µL. At this dilution, gluconic acid response was found to be linear in the range of 1–5 ppm. ^13^C gluconic acid at 2 ppm was used as internal standard and introduced in the analyte after filtering through 0.2-µ filter. For calibration, gluconic acid standards in the range of 1–5 ppm containing 2 ppm of internal standard in 1000 ppm glucose solution in mobile phase were prepared. During the analysis, the samples and calibrants were injected in triplicate and average response was used.

The chromatograms of glucose and gluconic acids for one of the samples of enzymatic hydrolysates have been shown in Additional file 1: Figure S1. Although gluconic and cellobionic acids coeluted with glucose and cellobiose, it was possible to accurately quantitate these acids by monitoring the area of extracted ion chromatograms of their molecular ions (sodium adducts). The ESI–MS experiments were carried out at the resolution in the range of 35,000 to 40,000 which ensured mass measurement accuracy up to the third place of decimal. Hence, the identification and quantitation of gluconic acid and cellobionic acid were unambiguous. With the Aminex HPX-87 H column, tri-saccharides are eluted first, followed by di-saccharides, mono-saccharides, formic acid, acetic acid, HMF, and furfural. Aldonic acids eluted with the corresponding sugars, with cellobionic acid eluting at 7.5 min and gluconic acid at 9.0 min, with an overall analysis time of approximately 11 min.

For quantitative analysis, isotope correction was made for isotopic abundance of 1.11% ^13^C in ^12^C. Therefore, 6.6% of the peak area of gluconic acid (*m/z* = 219.0475) was subtracted from that of the IS (*m/z* = 220.0508), and the ratio of gluconic acid to internal standard against gluconic acid concentration was plotted for calibration. The coefficient of determination (*R*^2^) of gluconic acid in the range of 1 to 5 ppm was found to be 0.986 which adequately established linearity of the response (Additional file 1: Figure S2). It was not possible to source ^13^C or deuterium-labeled cellobionic acid, and therefore, cellobionic acid was estimated assuming its response factor to be identical with gluconic acid. The results though not accurate were considered indicative.

To quantitate LPMO-mediated gluconic acid production, differential gluconic acid and cellobionic acid measurements were done according to the formula given below:

$$\begin{aligned} {\text{Differential}}\,{\text{gluconic}}\,{\text{acid (dGlcA)}} & = {\text{Gluconic}}\,{\text{acid}}\,{\text{concentration}}\,{\text{measured}}\,{\text{in}}\,{\text{the}}\,{\text{presence}}\,{\text{of}}\,{\text{ascorbate}} \hfill \\ & \quad - {\text{Gluconic}}\,{\text{acid}}\,{\text{concentration}}\,{\text{measured}}\,{\text{in}}\,{\text{the}}\,{\text{absence}}\,{\text{of}}\,{\text{ascorbate}} \hfill \\ \end{aligned}$$

$$\begin{aligned} {\text{Differential}}\,{\text{cellobionic}}\,{\text{acid (dClbA)}} & = {\text{Cellobionic}}\,{\text{acid}}\,{\text{concentration}}\,{\text{measured}}\,{\text{in}}\,{\text{the}}\,{\text{presence}}\,{\text{of}}\,{\text{ascorbate}} \hfill \\ & \quad - {\text{Cellobionic}}\,{\text{acid}}\,{\text{concentration}}\,{\text{measured}}\,{\text{in}}\,{\text{the}}\,{\text{absence}}\,{\text{of}}\,{\text{ascorbate}} \hfill \\ \end{aligned}$$

## Results and discussion

### Process scheme

**Fig. 1:**
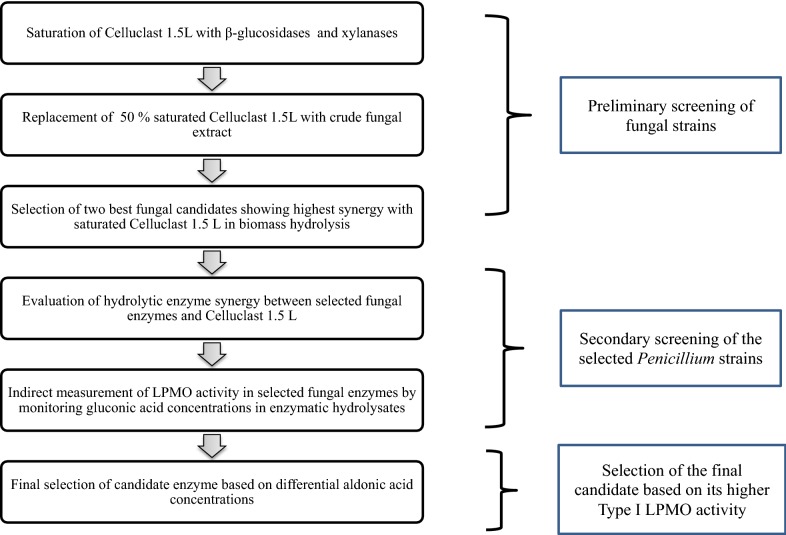
Process scheme. Schematic representation of the screening strategy applied to the screening of LPMO producing fungal strains

The main aim of this study was to devise a screening strategy that could be used to screen fungal enzymes for AA9 LPMO production. The screening strategy can be viewed in the process scheme, shown in Fig. 1.

### Preparation of saturated Celluclast 1.5L blend

The main objective of this study was to develop a screening protocol for fungal enzymes for AA9 LPMO production. The identification scheme adopted in this study exploits the synergistic interactions of cellulases and accessory enzymes involved in lignocellulosic biomass degradation. The screening strategy employed a saturated cellulase cocktail to identify potential synergistic proteins present in the fungal extract that might help in increasing biomass hydrolysis activity of this saturated cellulase cocktail.

A versatile and abundant lignocellulose biomass, rice straw, has been utilized here to screen and identify LPMO production ability of fungal candidates. The complex and heterogeneous composition of rice straw (Additional file 1: Table S1) served as an action platform for accessory enzymes to exhibit their synergistic interactions with cellulases, which helped in screening the enriched fungal secretomes.

Celluclast 1.5L was developed decades ago by Novozyme, but lacks several enzymatic components compared to its current Cellic^®^ variants. Several reports have shown that β-glucosidase, xylanases, and LPMOs are key boosters of cellulases and the exogenous addition of these enzymes can enhance the hydrolytic activity of Celluclast 1.5L [21, 22]. Notably in Celluclast 1.5L, nonhydrolytic accessory enzymes such as LPMOs are present in negligible amount [23]. Therefore, Celluclast 1.5L was spiked with saturating amounts of β-glucosidase and xylanase to maximize its hydrolysis efficiency, until saturation was achieved. Later, fungal secretomes were mixed with this saturated cocktail to formulate a blend of fungal extracts with saturated Celluclast 1.5L. Hydrolysis of pretreated rice straw was done with this blended formulation to explore some accessory fungal enzymes other than saturated hydrolases (β-glucosidase and xylanase), such as nonhydrolytic LPMOs.

**Fig. 2:**
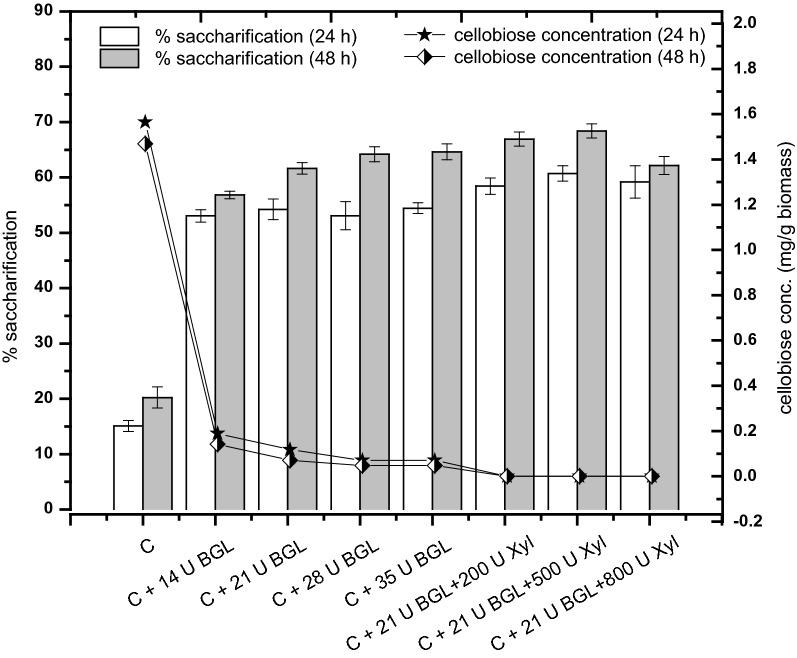
Saturation of Celluclast 1.5L with β-glucosidase and xylanases. Enzymatic blending was done for saturation of Celluclast 1.5L with different concentrations of β-glucosidase and xylanases. Minimum saturating concentrations of β-glucosidase enzymes suitable for 7 FPU of Celluclast 1.5L were defined based on depletion of cellobiose concentrations in the hydrolysates. After β-glucosidase saturation, xylanases were also blended to determine the minimum saturating xylanase concentration. 500 U of xylanase concentration was optimal to achieve saturation. The effect of enzyme supplementation was evaluated by recording % saccharification of pretreated rice straw at 24 h (white bars) and 48 h (gray bars) of hydrolysis using various enzymatic combinations. The error bars represent the standard deviation of three independent experiments. Symbol C represents Celluclast 1.5L (7 FPU), BGL represents commercial β-glucosidase enzyme (Novozymes 188), and Xyl represents commercial xylanase enzyme (Cellic^®^ HTec3)

Tertiary combinations of Celluclast 1.5L, β-glucosidase, and xylanase enzymes were evaluated for efficient hydrolysis of pretreated rice straw as shown in Fig. 2. Addition of 21 U/g biomass of β-glucosidase in 7 FPU of Celluclast 1.5L increased biomass hydrolysis by ~ 35% and further additions of β-glucosidase did not substantially increase the glucose yield. After β-glucosidase saturation, xylanase (200–800 U/gm biomass) supplementation in the cocktail was done, and it was observed that 500 U of xylanases/gm biomass were optimal in saturating Celluclast 1.5L. The effect of xylanase addition on the hydrolytic ability of saturated Celluclast 1.5L (~ 4%) was not profound as β-glucosidase addition. The absence of significant boosting effect in xylanase addition might be related to the low hemicellulose content in pretreated solids. Hence, a benchmark cocktail was selected for further hydrolysis experiments which contained 7 FPU of Celluclast 1.5L saturated with 21 U/g biomass of β-glucosidase and 500 U/g biomass of xylanases, and was termed as “saturated cocktail**.”**

### Screening of fungal strains

All the fungal strains were cultured in a corn steep liquor-based enriched media to produce enzymatic cocktails. The abundant micronutrients of this complex culture medium supported prolific fungal growth and it has also helped in inducing higher cellulase production [24].

Fungal enzymes were harvested after 96 h of fermentation, and their hydrolytic activities were measured against a variety of substrates: filter paper, carboxymethyl cellulose (CMC), *p*-nitrophenol from *p*-nitro phenyl-β-d-glucoside (pNPG) as shown in Table 1. The maximum filter paper activity of 7.23 IU/mL was observed in secretome of DBT-IOC-P-11-31, a mutant derived from *Penicillium funiculosum*. DBT-IOC-EU1 surpassed all its fellow mutant strains with a higher FPU of 4.08 IU/mL while its parent strain *Penicillium janthinellum* NCIM 1171 had a very low FPU of 0.87 IU/mL. Increase in FPU activity in mutants also correlated with a higher amount of extracellular protein secretion which infers that random mutagenesis of fungal strains helped in increasing enzyme production. Apart from FPU activities, mutants were also improved in terms of other enzymatic activities compared to their respective parent strain, and some of these were comparable to Celluclast 1.5L (such as CMCase and β-glucosidase activities of DBT-IOC-P-11-31, DBT-IOC-EU1 in Table 1). After determination of enzymatic activities on model substrates (Table 1), all these fungal strains were further evaluated for their ability to hydrolyze lignocellulosic biomass.

**Table 1:** Enzymatic activities determined in cellulase preparation used in this study

Enzyme	Protein (mg/mL)	Filter paper activity (FPU/ml)	CMC (IU/mL)	BGL (IU/mL)
*P. funiculosum* (NCIM 1228)	6.04 ± 0.09	2.13 ± 0.24	24.51 ± 0.24	23.29 ± 0.05
DBT-IOC-P-11	14.64 ± 0.29	5.66 ± 0.02	41.05 ± 1.81	42.51 ± 0.08
DBT-IOC-P-11-9	10.88 ± 0.19	5.51 ± 0.16	33.11 ± 0.81	48.53 ± 0.02
DBT-IOC-P-11-10	10.10 ± 0.29	6.37 ± 0.01	34.42 ± 0.23	53.04 ± 0.05
DBT-IOC-P-11-15	11.16 ± 0.19	3.31 ± 0.16	24.13 ± 1.04	49.96 ± 0.07
DBT-IOC-P-11-22	7.49 ± 0.90	6.76 ± 0.58	33.79 ± 1.60	62.52 ± 0.31
DBT-IOC-P-11-31	17.19 ± 0.38	7.23 ± 0.43	43.77 ± 0.02	75.88 ± 0.22
DBT-IOC-P-11-33	11.37 ± 0.14	4.05 ± 0.17	37.41 ± 1.15	54.24 ± 0.43
DBT-IOC-P-11-37	17.98 ± 0.23	4.41 ± 0.37	37.11 ± 0.91	40.65 ± 0.05
*P. janthinellum (*NCIM 1171)	13.58 ± 0.41	0.87 ± 0.06	21.45 ± 1.69	1.65 ± 0.05
DBT-IOC-EMS	10.40 ± 0.38	3.37 ± 0.14	30.54 ± 1.14	3.92 ± 0.02
DBT-IOC-EU1	10.22 ± 0.16	4.08 ± 0.23	50.38 ± 1.52	5.38 ± 1.08
DBT-IOC-EU4	11.95 ± 0.23	3.32 ± 0.09	33.56 ± 1.07	2.97 ± 0.02
DBT-IOC-EU1-DU3	10.22 ± 0.24	3.66 ± 0.14	49.08 ± 0.39	4.5 ± 0.01
*Trichoderma* RC-30	20.49 ± 0.51	5.77 ± 0.33	33.22 ± 0.16	3.79 ± 0.04
Celluclast 1.5L	184.82 ± 4.73	55.60 ± 2.50	45.77 ± 1.15	15.78 ± 0.05
Cellic^®^ Ctec2	220.45 ± 3.21	136.66 ± 1.12	ND	ND

*ND* not determined

**Table 2:** Biomass hydrolysis profiles of enzymes and their blended preparations

Legend	Enzyme	% Saccharification (24 h)	% Enhancement
Saturated cocktail	Celluclast 1.5L (7 FPU) + BGL (21U) + Xylanase (500 U)	59.68 ± 2.38	–
Base cocktail	Celluclast 1.5L (3.5 FPU) + BGL (21U) + Xylanase (500 U)	–	–
P”	Base cocktail + *P. funiculosum* (3.5 FPU)	60.15 ± 3.44	0.47
P-11”	Base cocktail + DBT-IOC-P-11 (3.5 FPU)	67.92 ± 1.72	8.24
P-11-9”	Base cocktail + DBT-IOC-P-11-9 (3.5 FPU)	62.20 ± 0.32	2.52
P-11-10”	Base cocktail + DBT-IOC-P-11-10 (3.5 FPU)	65.10 ± 0.53	5.42
P-11-15”	Base cocktail + DBT-IOC-P-11-15 (3.5 FPU)	65.18 ± 0.21	5.5
P-11-22”	Base cocktail + DBT-IOC-P-11-22 (3.5 FPU)	66.09 ± 0.38	6.41
P-11-31”	Base cocktail + DBT-IOC-P-11-31 (3.5 FPU)	68.07 ± 1.84	8.39
P-11-33”	Base cocktail + DBT-IOC-P-11-33 (3.5 FPU)	61.83 ± 2.38	2.15
P-11-37”	Base cocktail + DBT-IOC-P-11-37 (3.5 FPU)	64.80 ± 1.61	5.12
NCIM 1171”	Base cocktail + *P. janthinellum* (3.5 FPU)	61.36 ± 2.38	1.68
EMS”	Base cocktail + DBT-IOC-EMS (3.5 FPU)	60.46 ± 1.07	0.78
EU1”	Base cocktail + DBT-IOC-EU1 (3.5 FPU)	64.9 ± 2.90	5.22
EU4”	Base cocktail + DBT-IOC-EU4 (3.5 FPU)	58.3 ± 2.95	ND
EUI-DU3”	Base cocktail + DBT-IOC-EU1-DU3 (3.5 FPU)	60.5 ± 0.75	0.82
RC-30”	Base cocktail + RC-30 (3.5 FPU)	66.25 ± 1.1	6.57

*ND* enhancement was not detected

* Fungal extract spiking was done in base cocktail which contains Celluclast 1.5L (3.5 FPU) + BGL (21U) + Xylanase (500 U) to maintain constant enzyme dosage in terms of filter paper units FPU (7 FPU/g biomass) in all experiments

” Symbol represents the blend of fungal extract (3.5 FPU) and Celluclast 1.5L (3.5 FPU) employed to degrade pretreated rice straw

The screening of fungal strains was based on their ability to enhance biomass hydrolysis ability of commercial Celluclast 1.5L, which is a poor source of LPMOs. Many studies have adopted this screening strategy to identify fungal strains with beneficial enzymatic components [25, 26]. The present screening methodology is influenced by the similar approach, however the saturation of Celluclast 1.5L cocktail was done to explore new enzymes from fungi which have not been characterized yet through this screening strategy (such as new accessory enzymes). For the screening of fungal enzymes, the Celluclast 1.5L in “saturated cocktail” was replaced by 50% of candidate enzyme (fungal extracts). Therefore, blending of fungal extracts was done in such a way that 3.5 FPU of Celluclast 1.5L of the “saturated cocktail” was replaced with an equivalent 3.5 FPU of candidate fungal extracts as shown in Table 2.

**Fig. 3:**
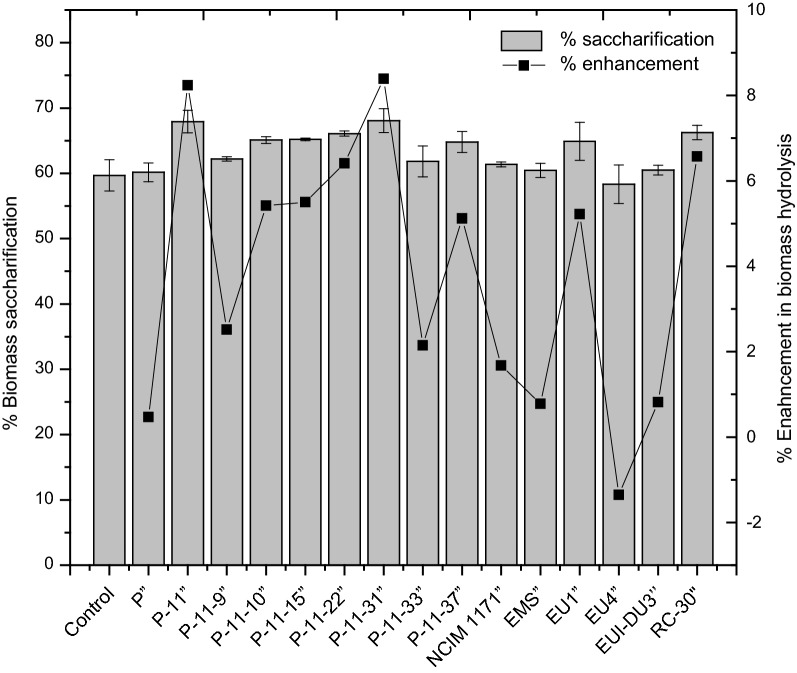
Biomass hydrolysis enhancement potential of fungal strains. Enhancements were observed in the hydrolysis of pretreated rice straw by means of Celluclast-fungal enzymatic blend. The control hydrolysis reaction was done by biomass hydrolysis using a saturated cocktail containing 7 FPU of Celluclast 1.5L + 21 U β-glucosidases + 500 U xylanases. Other reactions marked as (X”) represent screening cocktails which contain saturated Celluclast 1.5L blended with respective fungal enzymes. Hydrolysis reactions was carried out for 24 h, and the error bars represent the standard deviation of two independent experiments

Several enzymatic blends have displayed enhancement in biomass hydrolysis compared to the saturated cocktail. However, some mutants have exhibited a substantial enhancement in biomass hydrolysis yield such as P-11,” P-11-31,” and EU1” (8.24%, %, 8.39% and 5.22% respectively) compared to the saturated Celluclast cocktail (Fig. 3). It was interesting to note that the fungal extract of *RC*-*30* also enhanced the hydrolysis efficiency of Celluclast 1.5L, even though both enzymes are sourced from *Trichoderma.* This finding has also been observed earlier [27] and the reason for these differences in two *Trichoderma* enzymes could be attributed to protected culture conditions, growth requirements, and/or strain characteristics of commercial *Trichoderma* involved in Celluclast 1.5L production. However, the enhancement observed in the *Penicillium funiculosum* blend (P-11” or P-11-31”) is higher than that for the *Trichoderma RC*-*30”* blend, which indicates that *Penicillium* spp. have the potential to act as a new industrial workhorse for cellulase production [28].

The enhancement observed in the hydrolysis yield by using fungal-Celluclast 1.5L blend was attributed to the presence of synergistic fungal proteins, which may also include nonhydrolytic LPMOs, since Celluclast 1.5L lacks any substantial LPMO activity. The screening strategy was aimed at eliminating the synergy of hydrolytic enzymatic components between enzymatic blends to the maximum possible extent. Some of the popularly known cellulase boosters (β-glucosidase and xylanases) were saturated in Celluclast 1.5L and the acting FPU was maintained as a constant in all hydrolysis experiments (i.e. 7 FPU/g biomass). Since blending experiments rely on mixing different combinations of enzymes, FPU was used as a convenient and rapid method to load equal amounts of cellulases in blending experiments, even though in practice there may be some variation in endoglucanase activity in different fungal mixtures. It was anticipated that the observed enhancement was the result of a combined action of some new accessory hydrolytic enzymes coupled with nonhydrolytic cellulase boosters in fungal extracts, possibly LPMOs.

To further evaluate the effects of all possible enzymatic scenarios that may have contributed to the enhanced biomass hydrolysis yields, the detailed characterization of two fungal candidates from each *Penicillium* sp., DBT-IOC-P11 and DBT-IOC-EU1, was done, since they exhibited the highest hydrolysis enhancement in their respective groups.

Initially, the synergy of hydrolytic components in Celluclast-fungal enzymatic blends were analyzed to investigate the role of accessory hydrolytic enzymes in increasing biomass hydrolysis. Later, to analyze the contribution of LPMO enzymes in increasing biomass hydrolysis yield, the detection of LPMO enzymes was carried out using an Amplex Red assay coupled with gluconic acid measurements in enzymatic hydrolysates.

### Role of enzyme synergy in hydrolysis enhancement of strains

It has been shown in various studies that the enhancement in biomass saccharification depends on the synergy between different enzymes in the blend [29–31]. Hence, a comparative analysis of enzymatic blends was done to determine possible synergistic effects of hydrolytic enzymes that could be responsible for the strain’s enhancement potential. Enzymatic assays were performed to determine substrate hydrolytic activity, with calculations for total volume of enzymatic blend acting per gram of biomass.

Compared with the saturated cocktail, enzymatic activities for the P-11” blend was higher in all assays, which is consistent with higher biomass hydrolysis potential of this blend (Table 3). Recorded FPAse, CMCase, β-glucosidase, and protein content values for the P-11 blend were higher than those for the other two cocktails (Saturated cocktail and EU1”).

**Table 3:** Enzymatic activity of blend used for pretreated rice straw hydrolysis. Calculations were done for the enzymatic blend action per gram of biomass

Enzyme	Protein (mg/mL)	Filter paper activity (FPU/mL)	CMCase activity (IU/mL)	β-glucosidase activity (IU/mL)
Saturated cocktail	55.62 ± 1.38	11.5 ± 1.49	238.09 ± 2.08	48.32 ± 1.73
P-11”	48.67 ± 0.29	12.66 ± 1.3	254.41 ± 1.41	59.97 ± 1.16
EU1”	46.35 ± 0.28	12.54 ± 1.4	176.29 ± 3.5	50.9 ± 1.98

Saturated cocktail represents 7 FPU of Celluclast 1.5L saturated with 21 Units of β-glucosidase enzyme and 500 Units of Xylanases

**”** Symbol represents the blend of fungal extract (3.5 FPU) + Celluclast 1.5L (3.5 FPU) which was employed to degrade pretreated rice straw

Although the percentage enhancements of biomass hydrolysis by DBT-IOC-EU1 enzyme blend (EU1”) was approximately 5% higher than that of the saturated cocktail, the enzymatic activities of this blend was comparable to that of the saturated cocktail, while its CMCase activity was lower than that of the saturated cocktail (Table 3).

Some nonhydrolytic synergistic proteins, such as LPMOs, might have played a role in increasing the hydrolysis yield of strain DBT-IOC-EU1, as these enzymes are not detected in the model substrate hydrolysis assays reported in Table 3 [22]. Therefore, the next step was to detect the presence of LPMO enzymes in these fungal extracts that could enhance the biomass hydrolysis in these blends, compared to the saturated cocktail.

It is interesting to note that in the saturated cocktail the protein loading is higher than for both fungal blends, (P-11” and EU1”) although the acting FPU of blends were higher than saturated cocktail. This observation indicates that while carrying out biomass hydrolysis, enzymatic blending should be done in terms of FPU or BHU (biomass hydrolysis units) compared to protein loading, so that acting lignocelluloytic enzymes are blended in approximately equal amounts.

### Amplex Red assay of fungal enzymes

Later, culture supernatants of selected *Penicillium* strains were evaluated for hydrogen peroxide production using an Amplex^®^ Red assay. Ascorbate supplemented Amplex^®^ Red dye was employed to characterize the H_2_O_2_ production ability of fungal strains. The assay was carried out in the presence of 300 µM ascorbate as electron donor to characterize the LPMO activity of fungi. In order to determine other sugar-oxidizing enzymes present in fungal strains, total H_2_O_2_ production ability of the fungal enzymes was measured in the presence of ascorbate, glucose, and cellobiose, following a previously described method [32].

**Fig. 4:**
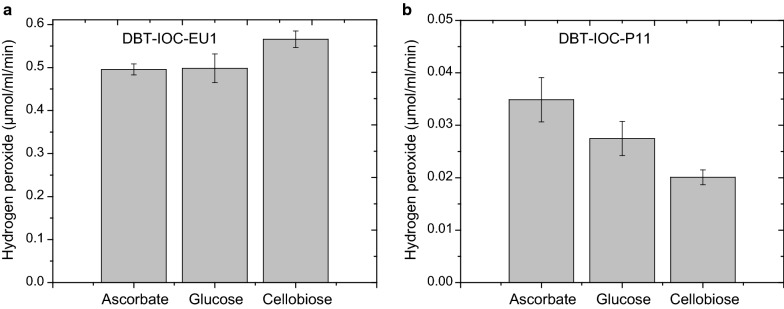
Hydrogen peroxide production capability of fungal enzymes. Amplex^®^ Red assay was performed in the presence of ascorbate, glucose, and cellobiose. The activities have been expressed as specific activity, i.e., μmol/mL/min H_2_O_2_ released per mg of fungal protein harvested at 96 h of fermentation. The error bars represent the standard deviation of the three independent experiments. Left panel shows the Amplex^®^ Red activity of DBT-IOC-EU1, and right panel contains oxidative activity of DBT-IOC-P-11

This assay revealed that mutant DBT-IOC-EU1 has remarkably high Amplex^®^ Red activity, which was nearly tenfold higher than that for strain DBT-IOC-P-11 (Fig. 4). Addition of cellobiose as well as glucose was able to induce slightly higher production of H_2_O_2_ compared to an ascorbate supplemented reaction mixture. Whereas, in *P. funiculosum* mutant (DBT-IOC-P-11) the ascorbate containing reaction assay produced highest amount of H_2_O_2_, most likely due to the presence of active LPMOs in *P. funiculosum* strain.

Although both strains belong to the same genus, the significant difference in H_2_O_2_ production capability indicated different roles of these strains in lignocellulose degradation. This assay indicated a lower level of oxidases for strain *P. funiculosum* compared to *P. janthinellum*. Due to the presence of various H_2_O_2_ producing oxidases in crude enzymes the Amplex^®^ Red assay is not specific for determining LPMO activity of crude cellulases, but provides an initial useful simple screening test for LPMO-like activity that results in hydrogen peroxide production, particularly when applying ascorbate-induced activity. In our case, the ascorbate-induced activity of strain P-11 was higher than that of EU1, which is consistent with results from the LC–MS assay and is consistent with relative LPMO activity levels.

Furthermore, both *Penicillium* strains were examined for the presence of cellobiose dehydrogenase (CDH) activities using DCPIP as the electron donor, and it was observed that none of the extracts showed any substantial cellobiose dehydrogenase activity. Although the draft genome sequence of *Penicillium janthinellum* ATCC 10455 v1.0 confirmed the presence of one CDH gene in this species (https://genome.jgi.doe.gov/) and a proteomic study of *Penicillium funiculosum* indicated the secretion of one AA3 GMC oxidoreductases [33], none of the fungal enzymes in this study, however, displayed any substantial CDH activity. These findings can be attributed to the use of Avicel as a carbon source in the submerged culture, which might not be able to induce enough levels of oxidases in fungal strains. Therefore, to analyze the effect of LPMOs on biomass hydrolysis enhancements, gluconic acid concentrations were measured in enzymatic hydrolysates of these strains using LC–MS.

### Quantitation of oxidative activities of enzyme using LC–MS

The hydrolysis of pretreated rice straw was carried out at a higher substrate loading of 10% dosed with 7 FPU of *Penicillium janthinellum* and *Penicillium funiculosum* mutant, along with Cellic^®^ Ctec2 as control. The reactions were carried out without any external electron donor such as ascorbate, since it was anticipated that the insoluble fractions of pretreated biomass act as electron donor for LPMOs [34]. The analysis was done for hydrolytic as well as oxidative activities of test enzymes by quantitating glucose as well as gluconic acid released in the reaction hydrolysates. Glucose concentration was measured with the help of HPLC and quantitation of gluconic acid was done by using LC–ESI–MS for superior selectivity and sensitivity. Due to the lack of C4 oxidation standards, only Type I LPMO activity was measured through gluconic acid quantitation in hydrolysates. Since the product distribution studies of LPMO enzymes (such as the quantitation of oxidized cello-oligomers produced in the reaction) is not the main aim of this study, quantitation of Type I LPMO activity was carried out by evaluating only the gluconic acid concentration in hydrolysates. Previous studies have demonstrated that gluconic acid concentrations can be attributed to the activity of C1 LPMOs present in *Trichoderma*-based commercial cellulases. However, it is worthwhile noting that the lack of CDH activity in these commercial cellulases has made this method applicable to *Trichoderma*-based commercial enzymes, it might not be the case for other enzymatic systems.

In previous reports where gluconic acid was measured in Cellic^®^ Ctec2, it was demonstrated that the production of gluconic acid is dependent on various parameters (Table 4). Different process strategies significantly affect the LPMO activity such as hydrolysis temperature, biomass loading, different pretreatment technologies, and different substrates significantly influence the LPMO activity. Hence, substrate specific profiling of cellulases shall be carried out before applying LPMOs in biofuel production on industrial scale.

**Table 4:** Comparative analysis of different lignocellulose hydrolysis conditions yielding gluconic acid after cellulose oxidation Cellic^®^ Ctec2 at 50 °C

Biomass	Pretreatment method	% Biomass loading	Hydrolysis period	% Hydrolysis	% Glucose oxidation	References
Wheat straw	Hydrothermal	30	144 h	85	4.1	[14]
Filter paper	–	5	108 h	83	0.7	[14]
Wheat straw	Hydrothermal	30	72	63	2.5	[35]
Corn Stover	Hydrothermal	15	96 h	50	0.49	[16]
Bagasse	–do–	15	–do–	87	0.43	[16]
Bagasse	Organosolv	15	–do–	60	0.12	[16]
Avicel*	–	5	72 h	53	1.89	[36]
Rice straw	Dilute acid	10	48 h	80	0.30	This study

* This Avicel hydrolysis reaction was incubated with sugarcane bagasse lignin-derived compounds which facilitated enhanced LPMO activity of Cellic^®^ Ctec2

The highest glucose and gluconic acid concentrations measured in the hydrolysates of pretreated rice straw were observed for the Cellic^®^ Ctec2 enzyme. At 48-h, 80.48% biomass hydrolysis was recorded for Cellic^®^ Ctec2 enzyme, with a concomitant production of 0.170 g/L of gluconic acid, which accounts for 0.3% oxidation of total glucose released in the hydrolysate. To the best of our knowledge, this is the first report to quantitate Type I LPMO activity of Cellic^®^ Ctec2 on dilute acid-pretreated rice straw, and the biomass pretreatment and hydrolysis conditions in this present study are quite different from those used in earlier studies (Table 4) which makes it difficult here to compare best LPMO yielding reaction conditions. However, % cellulose oxidation potential of the Cellic^®^ Ctec2 enzyme reported here falls in the range of earlier reported cellulose oxidation values for the same enzyme, and hence supports the suitability of sensitive LC–MS quantitation of LPMOs as an alternative to HPAEC-PAD measurements. Although not a high throughput method, the LC–MS is complete in 11 min, compared with about 20 to 30 min for the established HPAEC-PAD method, since the elution times for gluconic acid and cellobionic acid are about half for the LC–MS method. The LC–MS method also has higher sensitivity than the HPAEC-PAD method, and so is a useful alternative for low concentrations of gluconic acid and cellobionic acid that are normally present in enzymatic hydrolysates derived from crude fungal extracts.

**Fig. 5:**
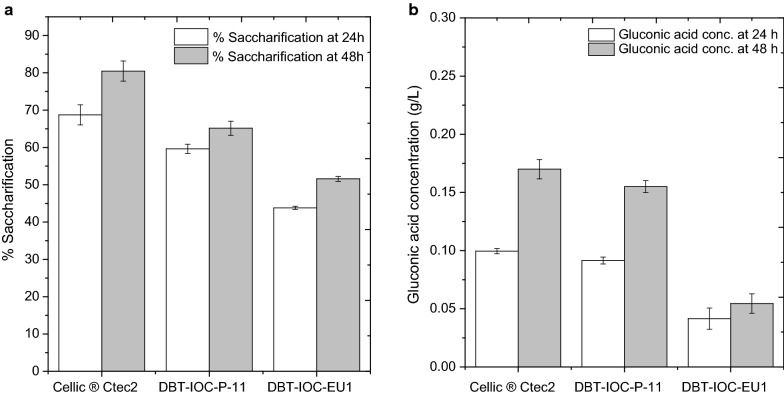
Saccharification of pretreated rice straw carried out with selected fungal strains along with commercial enzyme Cellic^®^ Ctec2. Cellulase enzyme loading was 7 FPU/g biomass. **a** Left pane contains hydrolysis data recorded as % glucan conversion at 24 h (white bars) and 48 h (gray bars). **b** Glucose oxidation has been quantitated in enzymatic hydrolysates of pretreated rice straw enzymes using ESI–LC–MS. The data have been recorded at 24 h (white bars) and 48 h (gray bars) of hydrolysis. The error bars represent the standard deviation of three independent experiments

Saccharification ability of the *P. funiculosum* mutant DBT-IOC-P-11 was 65.15%, while the biomass saccharification potential of *P. janthinellum* mutant DBT-IOC-EU1 was 51.57%. The glucan conversion ability of both fungal enzymes was lower than that of Cellic^®^ Ctec2, although for glucose oxidation the DBT-IOC-P-11 enzyme had comparable oxidative activity to the commercial enzyme (Fig. 5). Gluconic acid concentrations in DBT-IOC-P-11 hydrolysates were 0.155 g/L which accounts for 0.33% cellulose oxidation, which was slightly higher than the 0.30% cellulose oxidation of the commercial enzyme Cellic^®^ Ctec2. On the other hand, DBT-IOC-EU1 hydrolysates contained lower amounts of gluconic acid (0.054 g/L), which corresponded to a lower cellulose oxidation ability (0.16%), compared to DBT-IOC-P-11 and Cellic^®^ Ctec2 (0.33% and 0.30% cellulose oxidation respectively).

If the observed trend of gluconic acid concentrations in hydrolysates correlates to the Type 1 LPMO activity of fungal enzymes then it can be inferred that *P. funiculosum* mutant DBT-IOC-P-11 has higher LPMO activity than strain DBT-IOC-EU1, which may result in enhanced biomass hydrolysis of pretreated rice straw when it was blended in saturated cocktail of Celluclast 1.5L. It is also interesting to note that the growth and production media employed in this study induced higher cellulase enzyme’s secretion, which might have led to higher gluconic acid production in DBT-IOC-P-11 hydrolysates compared to the other enzymes. However, it is possible to overestimate gluconic acid values as Type I LPMO activity indicators in crude enzymes, if extracts of fungi contain some other aldonic acid producing oxidases (such as cellobiose dehydrogenase or glucose oxidase) along with LPMOs. To avoid the overestimation of Type I LPMO activity in crude enzymes, ascorbate-mediated differential aldonic acid quantitation was done to evaluate actual LPMO activity of crude fungal enzymes.

### Differential aldonic acid quantitation helps in determination of Type I LPMO activities

To employ the aldonic acid/LPMO quantitation method for crude fungal extracts, which is the primary aim of this research, it was decided to analyze the hydrolysates with the help of extrinsic LPMO electron donor (ascorbate), and the actual LPMO activity for fungal enzymes was monitored through differential aldonic acid concentration produced after the exclusion of residual activity from ascorbate negative controls. In order to quantitate the ascorbate-mediated LPMO activity of the enzymes, differential gluconic acid (d-GlcA) and cellobionic acid (d-ClbA) measurements were carried out to quantitate C1 LPMO enzymatic activity. For this purpose, enzymatic hydrolysis of Avicel was carried out with enzyme extracts from strain DBT-IOC-P-11, DBT-IOC-EU1 and commercial enzyme Cellic^®^ Ctec2, blended with saturating amounts of β-glucosidase (21U). Hydrolysis reactions were set up in the presence and the absence of ascorbic acid (1 mM), and the hydrolysates from different hydrolysis conditions to produce gluconic and cellobionic acid were analyzed. The use of Avicel in this comparative set of ascorbate aimed at excluding interfering activity from other oxidases (such as cellobiose dehydrogenases or glucose oxidase), which could have been overestimated while quantitating C1 LPMO activity of enzymes in the pretreated rice straw hydrolysates (Fig. 5).

**Fig. 6:**
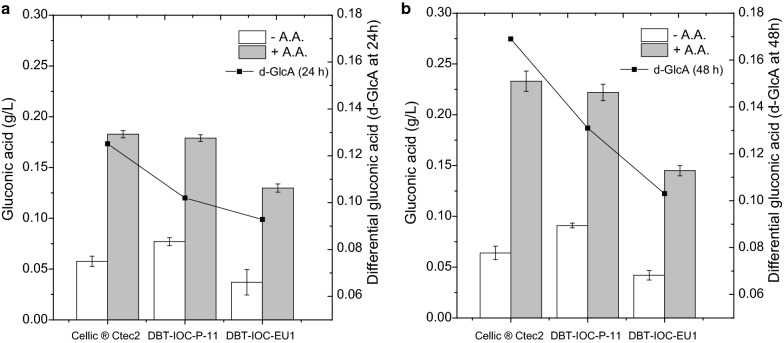
Gluconic acid production in Avicel hydrolysates. The measured gluconic acid concentrations were produced in the absence (white bars) and the presence (gray bars) of 1 mM ascorbic acid. Avicel hydrolysis was carried out with 7 FPU of cellulases saturated with 21 U of β-glucosidases. **a** Gluconic acid concentration of Avicel hydrolysates recorded at 24 h and **b** gluconic acid concentrations at 48 h of hydrolysis reactions. Differential gluconic acid concentrations (d-GlcA) represent gluconic concentrations (g/L) of hydrolysates produced only in the presence of ascorbate that has been indicated with the help of black solid lines in secondary *Y*-axis. The error bars represent the standard deviation of three independent experiments

**Fig. 7:**
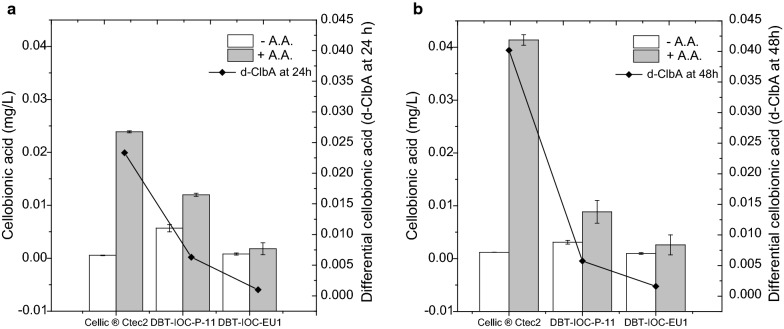
Cellobionic acid production in Avicel hydrolysates. The measured cellobionic acid concentrations were produced in the absence (white bars) and the presence (gray bars) of 1 mM ascorbic acid. Avicel hydrolysis was carried out with 7 FPU of cellulases saturated with 21 U of β-glucosidases. **a** Cellobionic acid concentration (mg/L) of Avicel hydrolysates recorded at 24 h and **b** cellobionic acid concentrations at 48 h of hydrolysis. Differential cellobionic acids (d-ClbA) which represent cellobionic concentrations (mg/L) of hydrolysates produced only in the presence of ascorbate and has been indicated by means of black solid lines in secondary *Y*-axis. The error bars represent the standard deviation of the three independent experiments

After ascorbate addition, the concentration of C1-oxidized products (gluconic and cellobionic acid) was increased in the Avicel hydrolysates of all enzymes compared to their ascorbate negative control reactions. It was observed that the degree of enhancement in aldonic acids concentration was highest in Avicel hydrolysates of Cellic^®^ Ctec2. The differential gluconic acid (d-GlcA) and cellobionic acid (d-ClbA) measurements of Cellic^®^ Ctec2 was highest, followed by strain DBT-IOC-P-11, while the lowest activity was observed for strain DBT-IOC-EU1 (Figs. 6, 7).

At 48 h, the highest d-GlcA concentrations were observed for Cellic^®^ Ctec2 (0.169 g/L), followed by *P. funiculosum* DBT-IOC-P-11 hydrolysate (0.131 g/L), and strain DBT-IOC-EU1 (0.103 g/L), as shown in Fig. 6. The same trend has been observed for d-ClbA concentration in Cellic^®^ Ctec2 hydrolysate (0.041 mg/L or 41 ppb) whereas strain P-11 (0.00575 mg/L) had higher differential cellobionic acid values compared to strain EU1 (0.00161 mg/L), as show in Fig. 7.

After ascorbate supplementation, cellobionic acid accumulation was observed for 48 h. After ascorbate supplementation, cellobionic acid accumulated in Avicel hydrolysates, and the observed accumulation was the highest for Cellic^®^ Ctec2, followed by *P. funiculosum* P-11, and then *P. janthinellum* strain EU1. At 48 h, the cellobionic acid concentration of fungal hydrolysates did not increase substantially, however, in Cellic^®^ Ctec2 hydrolysate, the cellobionic acid values increased along with gluconic acid production. It is known that high cellobionic acid concentrations inhibit β-glucosidase activity [14, 37], and higher LPMO activity increases both gluconic and cellobionic acid concentrations. This could explain the higher cellobionic acid concentrations accumulated in the Cellic^®^ Ctec2 hydrolysates, due to its presumably higher LPMO activity. The observed LPMO activity for the fungal strains was comparatively lower than that for the commercial enzyme Cellic^®^ Ctec2, and hence, the observed cellobionic acid concentrations were lower in the fungal hydrolysates (Fig. 7b).

For fungal enzymes, accumulation in cellobionic acid concentration was higher for strain *P. funiculosum* mutant P-11 and was the least for *P. janthinellum* strain EU1 (Fig. 7). This observation implies that *Penicillium funiculosum* P-11 is rich in C1 LPMO fractions compared to strain EU1, according to the observed higher differential gluconic acid (d-GlcA) and cellobionic acid (d-ClbA) concentrations in hydrolysates of this strain.

Hence, it can be concluded that the secretome of the *P. funiculosum* mutant is not only enriched in hydrolases but also contains higher oxidative activities (such as LPMOs) than does *P. janthinellum*. The differential gluconic acid concentration indicated that Type I LPMO activity of this strain is higher, resulting in increasing biomass hydrolysis enhancement of pretreated rice straw when blended in saturated cocktails of Celluclast 1.5L.

Owing to the presence of hydrolases, as well as oxidases such as LPMOs, *P. funiculosum* has the potential to become an important lignocellulosic industrial strain and has the potential to replace *Trichoderma*-based commercial cellulase preparations.

## Conclusion

After a three-stage screening protocol and detailed LC–MS-based analysis of hydrolysates, it can be concluded that quantitation of LPMOs in a heterogeneous group of crude fungal extracts can be done accurately using differential gluconic acid concentrations. Absolute quantitations of gluconic acid concentrations are suitable to calculate Type I LPMO activity in *Trichoderma*-based commercial cocktails; however, in the analysis of natural and crude fungal extracts or complex cellulase cocktails, an LPMO-specific screening method is required. This ESI–MS method enables monitoring of the release of very low levels of oxidized gluco-oligosaccahride, so that the method is suitable as a screening method in natural microbes. In the absence of suitable methods for the quantitation of LPMOs, targeted screening in crude fungal extracts is challenging. The current LC–MS methods using quantitation of change in GlcA values from hydrolysates could be used as a screening tool. Applying this method to the screening of LPMOs from fungal extracts, it was shown that *P. funiculosum* may be a useful industrial cellulolytic strain, which contains oxidative LPMO enzymes and has the potential to replace *Trichoderma*-based cellulases in biorefinery applications.

## Additional file

**Additional file 1: Table S1.** Chemical composition of native and pretreated rice straw. **Figure S1.** Separation of sugars and aldonic acids in ESI–MS chromatogram. **A** Base peak chromatogram (top curve) was separated into extracted ion chromatogram of glucose (middle curve) and gluconic acid (bottom curve). **B** Total ion chromatogram (TIC) was further resolved by **C** extracted ion chromatograms of glucose (*m/z* = 203.0526), gluconic acid (*m/z* = 219.0475) in calibration mixture. **Figure S2.** Standard curve of gluconic acid with internal standard. *X* axis represents calibration standard solutions of gluconic acid (1–5 ppm) and each calibration solution was prepared as a mixture of gluconic acid (1–5 ppm) plus 2 ppm internal standard C^13^ gluconic acid plus 1000 ppm glucose standard. *Y*-axis represents the ratio of mass response or intensity of gluconic acid vs C^13^ gluconic acid whose *m/z* values are 219.0475 and 220.0508 respectively.

## Data Availability

All data generated or analyzed during this study are included in this published article.
