# Supplementary material for: A screening approach for assessing lytic polysaccharide monooxygenase activity in fungal strains
Source: Biotechnol Biofuels. 2019 Jul 22;12:185. doi: 10.1186/s13068-019-1526-4 (PMC6643308; doi:10.1186/s13068-019-1526-4)
Supplement: Supplementary file 1 — Additional file 1: Table S1. Chemical composition of native and pretreated rice straw. Figure S1. Separation of sugars and aldonic acids in ESI–MS chromatogram. A Base peak chromatogram (top curve) was separated into extracted ion chromatogram of glucose (middle curve) and gluconic acid (bottom curve). B Total ion chromatogram (TIC) was further resolved by C extracted ion chromatograms of glucose (m/z = 203.0526), gluconic acid (m/z = 219.0475) in calibration mixture. Figure S2. Standard curve of gluconic acid with internal standard. X axis represents calibration standard solutions of gluconic acid (1–5 ppm) and each calibration solution was prepared as a mixture of gluconic acid (1–5 ppm) plus 2 ppm internal standard C13 gluconic acid plus 1000 ppm glucose standard. Y-axis represents the ratio of mass response or intensity of gluconic acid vs C13 gluconic acid whose m/z values are 219.0475 and 220.0508 respectively. [file 13068_2019_1526_MOESM1_ESM.docx]

**Additional Table S1**

**Chemical composition of native and pretreated rice straw**

| **Components (%)** | **Native** | **Pretreated rice straw** |
| --- | --- | --- |
| Glucan | 36.9 | 70.6 |
| Xylan | 20 | 4.2 |
| Arabinan | 3.0 | 0.2 |
| Lignin | 13.1 | 21.1 |
| Ash | 6.3 | 4.0 |
| Acetic acid | 1.1 | 0.0 |
| Extractives | 19.8 | 0.0 |

**Figure S1**


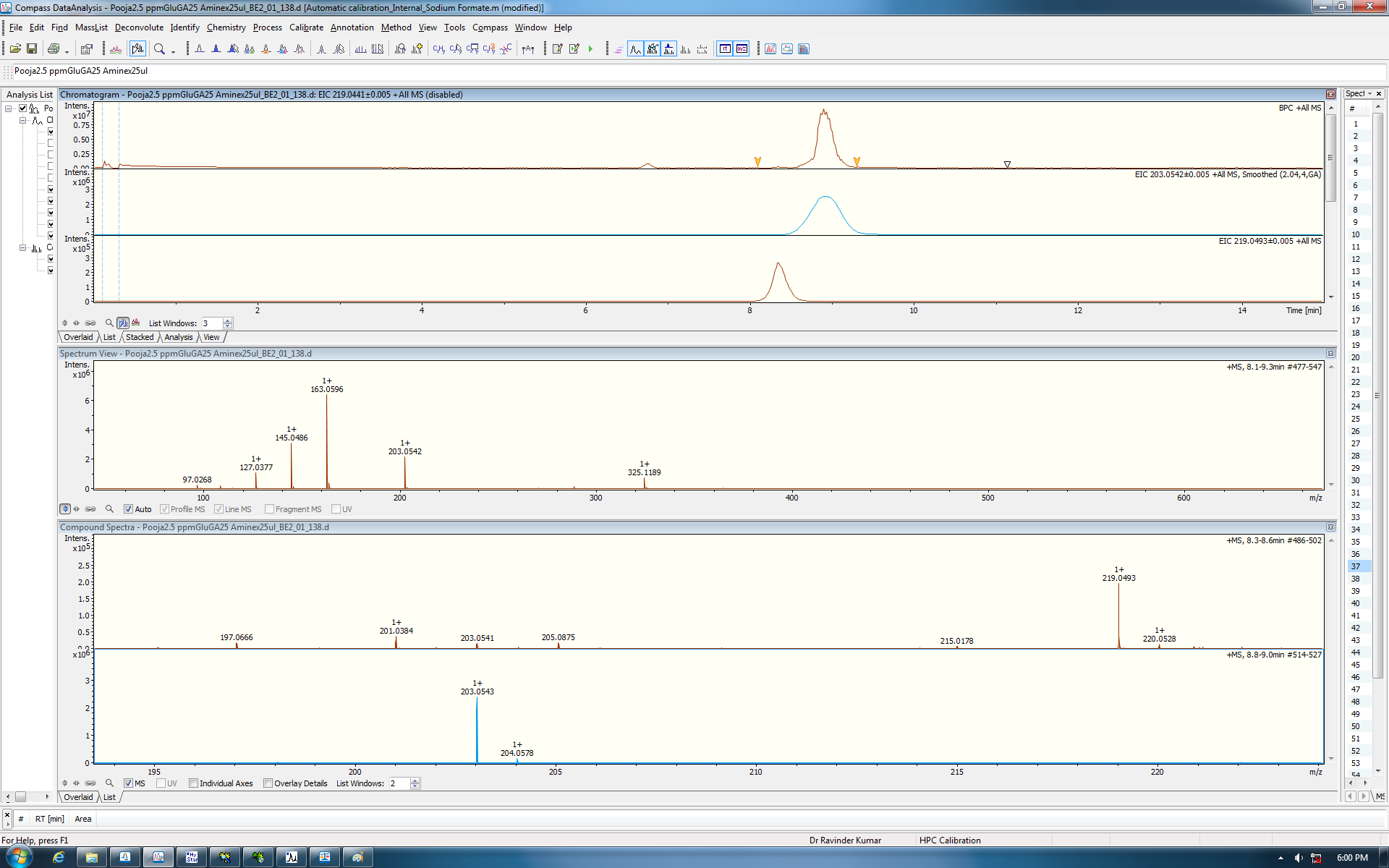


A

B

C

**Separation of sugars and aldonic acids in ESI-MS chromatogram.** (A) Base peak chromatogram (top curve) was separated using ion chromatogram of glucose (middle curve) and gluconic acid (bottom curve). (B) Total ion chromatogram (TIC) was further resolved by (C) extracted ion chromatograms of glucose (m/z = 203.0526) and gluconic acid (m/z = 219.0475) in calibration mixture .

**Figure S2**

*Figure S2.* **Standard curve of gluconic acid with internal standard.** The X axis represents the calibration standard solutions of gluconic acid (1-5 ppm) and each calibration solution was prepared as a mixture of gluconic acid (1-5 ppm) plus 2 ppm of internal standard ^13^C gluconic acid, plus 1000 ppm glucose standard. The Y – axis represents the ratio of mass response/intensity of gluconic acid vs ^13^C gluconic acid, whose m/z values are 219.0475 and 220.0508, respectively.
